# Exploring the Association Between Thyroid Density Assessed by Non-contrast Computed Tomography and Serum Thyroid-Stimulating Hormone (TSH) Levels in Hypothyroid Patients

**DOI:** 10.7759/cureus.48653

**Published:** 2023-11-11

**Authors:** Priyanka Chaudhary, Jaya Pamnani, Kirti Rana, Ashwani K Khandekar

**Affiliations:** 1 Radiodiagnosis, All India Institute of Medical Sciences, Raipur, IND; 2 Diagnostic and Interventional Radiology, Geetanjali Medical College and Hospital, Udaipur, IND; 3 Radiodiagnosis, Dr. Sampurnanand Medical College and Associated Group of Hospitals, Jodhpur, IND; 4 Radiodiagnosis, Geetanjali Medical College and Hospital, Udaipur, IND

**Keywords:** serum thyroid-stimulating hormone, euthyroid, hypothyroid, non-contrast computed tomography, thyroid density

## Abstract

Objective: This study aims to investigate the relationship between thyroid computed tomography (CT) density and serum thyroid stimulating hormone (TSH) levels in patients with hypothyroidism.

Methods: The research involved 60 hypothyroid patients aged 18 years and older, with TSH levels exceeding 5 mIU/mL or those already receiving thyroid supplementation therapy. These individuals had undergone non-contrast CT chest scans due to concurrent respiratory concerns. The thyroid CT densities, as evaluated through non-contrast chest CT, were compared to those of euthyroid patients with normal TSH levels. Correlation analysis was conducted to assess the relationship between serum TSH levels and thyroid CT densities.

Results: Hypothyroid patients exhibited significantly reduced thyroid CT densities (P<0.0001). The correlation analysis revealed an inverse correlation between TSH levels and thyroid CT densities in hypothyroid patients (r=-0.365, p=0.020) and a positive correlation (r=0.270, p=0.036) in euthyroid patients.

Conclusion: This study demonstrates a substantial correlation between thyroid CT density and serum TSH levels. As TSH levels increase in hypothyroid patients, thyroid CT density tends to decrease.

## Introduction

Hypothyroidism is a widely prevalent medical condition, with a higher occurrence in regions marked by iodine deficiency, impacting around 1% to 2% of the population [1]. Notably, this condition disproportionately affects women, with an incidence that is ten times higher than that in men. In the general population, hypothyroidism affects up to 9% of women and 1-2% of men [1].

In developed nations, hypothyroidism primarily arises from an underlying autoimmune mechanism, accounting for nearly 90% of non-iatrogenic cases [1]. However, a minority of cases may be linked to congenital factors, postpartum conditions, or the use of medications like glucocorticoids [2,3]. Autoimmune hypothyroidism can be classified into two primary types: primary atrophic hypothyroidism and hypertrophic autoimmune hypothyroidism [4,5]. The atrophic variant, initially described by Ord et al. in the late 1890s, is characterized by damage to the thyroid gland [6]. In 1912, Hashimoto introduced the hypertrophic variant, subsequently named Hashimoto's disease [7].

Thyroid gland volume ranges from 10 to 15 mL in females and 12 to 18 mL in males [8]. Iodine is incorporated into the thyroid gland follicles under the influence of thyroid-stimulating hormone (TSH) [9]. In the non-contrast computed tomography (NCCT) study, a normal thyroid gland exhibits high CT attenuation values, primarily due to the increased electron density of iodine molecules. In cases of hypothyroidism, the CT attenuation of the thyroid gland is notably lower than that of normal thyroid glands [10]. This reduced attenuation is a result of reduced iodine concentration in the thyroid follicles, decreased colloid content, and an increase in both follicular cells and interstitial structures. Therefore, a rapid visual assessment of thyroid attenuation can effectively identify patients with hypothyroidism, characterized by low attenuation of thyroid glands [10]. Consequently, the high serum TSH concentration in individuals with hypothyroidism is linked to this diminished thyroid attenuation [11,12].

Previous reports from Hoffer et al. and Patton et al. have suggested higher iodine content in diffuse toxic goiters, which is associated with increased CT attenuation values [12,13].

While previous research has raised the potential of CT density in assessing thyroid functional status, the precise relationship between CT density and thyroid gland function remains unexplored [8]. In our current study, we propose the hypothesis that hypothyroidism can be detected through reduced density in NCCT scans. Additionally, we aim to investigate whether reduced NCCT density of the thyroid gland correlates with elevated TSH levels in individuals with hypothyroidism. This research seeks to contribute to the understanding of the diagnostic potential of CT density in thyroid disorders.

## Materials and methods

This prospective study received approval from the Ethic Committee of Dr. Sampurnanand Medical College, Jodhpur (smnc/2017/83), and informed consent was obtained from all participants. The subject group consisted of a total of 60 individuals who met the inclusion criteria, including those with acquired hypothyroidism (TSH levels exceeding 5 mIU/mL) and hypothyroid patients undergoing thyroid supplementation therapy. These individuals were primarily referred for CT scans in the radiology department to assess recurrent or chronic respiratory symptoms. Exclusion criteria for the study encompass subjects who have previously undergone thyroid surgery or radioactive iodine therapy for known colloid goiter or thyroid malignancy, those with visibly enlarged thyroid glands or nodular goiters, and individuals using medications that can influence thyroid function, such as lithium, amiodarone, interferon-gamma, or steroids. Pregnant participants, confirmed through pregnancy testing, as well as women utilizing contraceptives, are excluded from the study. Additionally, individuals with a documented history of thyroid, lung, or paranasal sinus malignancies are not considered within the scope of the study.

The findings from the subject group of hypothyroid subjects were then compared to a control group comprising 60 euthyroid individuals who did not exhibit clinical signs of hypothyroidism and had normal TSH levels. These control subjects presented to the radiology department for CT chest scans due to their respiratory symptoms.

Detailed medical histories were gathered from each participant to identify any contraindications for CT scans. All participants underwent NCCT of the thorax, utilizing a PHILIPS Ingenuity core 64-slice multi-detector CT scanner. Post-processing interpretation was conducted using Philips windows workstation and Intellispace software. Given that this study employed imaging parameters consistent with a low-dose NCCT/HRCT protocol and included a supraclavicular assessment as part of a comprehensive chest examination, the thyroid gland was encompassed in the chest CT scans of the subjects.

Hounsfield measurements were used to determine thyroid gland density on the NCCT scans, with three to four regions of interest (ROI) selected in both thyroid lobes and the isthmus. The Hounsfield measurements were conducted by a radiologist with five years of experience in the field of radiology. The average CT density of all ROI locations was then recorded. Correlation analyses between TSH levels and CT attenuation were conducted using appropriate statistical methods in both the study (hypothyroid) and control (euthyroid) groups with the help of software (IBM SPSS Statistics, IBM Corp., Armonk, NY).

## Results

The study encompassed included a total of 60 hypothyroid subjects, comprising six males and 54 females, and an equivalent number of 60 euthyroid subjects, with 33 males and 27 females. Among the hypothyroid subjects, 60% were under the age of 50, whereas the euthyroid group exhibited an even distribution between individuals below 50 and those above 50 years of age. It is worth noting that a significant proportion of hypothyroid subjects, specifically 80% (n=48), were housewives, while the euthyroid group encompassed individuals with diverse occupations (Table 1).

**Table 1:** CT thyroid density of hypothyroid and euthyroid subjects *No of subjects. CT, computerized tomography; NCCT, non-contrast computed tomography

Thyroid density on NCCT	Hypothyroid		Euthyroid	
	n* (60)	%	n* (60)	%
≤50	5	8.33	0	0.00
50.1-70	22	36.67	0	0.00
70.1-90	12	20.00	11	18.33
90.1-110	9	15.00	21	35.00
110.1-130	5	8.33	13	21.67
>130	7	11.67	15	25.00

The mean thyroid density in hypothyroid subjects was 83.04 HU units, with a standard deviation of ±31, in contrast to euthyroid subjects, whose mean thyroid density was 111.84 HU units, with a standard deviation of ±23.10. The application of a Student's t-test revealed a statistically significant difference in the mean thyroid density values between hypothyroid and euthyroid subjects, with a p-value of <0.0001 and a t-value of 5.769 (Table 2).

**Table 2:** Correlation between TSH and CT thyroid density in hypothyroid and euthyroid subjects TSH, thyroid stimulating hormone; CT, computerized tomography

Correlation	Hypothyroid		Euthyroid	
	r-value	p-value	r-value	p-value
TSH v/s thyroid density	-0.365	0.020	0.270	0.036

Analysis of thyroid density distribution indicated that in the majority of hypothyroid subjects (36.67%, n=22), the density fell within the range of 50.1-70 HU units, while in the majority of euthyroid subjects (35%, n=21), it was within the range of 90.1-110 HU units. Notably, none of the euthyroid subjects exhibited a thyroid density of ≤70 HU units.

Out of the total 60 hypothyroid subjects, serum TSH reports were available for 40 individuals. Among these, only four had a TSH level below 5 mIU/mL, while the remaining 36 subjects had TSH levels exceeding 5 mIU/mL. For hypothyroid patients with TSH levels below 5 mIU/mL, 25% had thyroid density below 90 HU, and 75% had density above 90 HU. In contrast, for hypothyroid patients with TSH levels at or above 5 mIU/mL, 72.2% had thyroid density below 90 HU, and 27.77% had density above 90 HU. This suggests that a majority of patients with TSH levels below 5 mIU/mL exhibited thyroid density above 90 HU, while those with TSH levels at or above 5 mIU/mL displayed thyroid density below 90 HU (Table 3).

**Table 3:** Comparison of thyroid density with TSH level in hypothyroid subjects *No of subjects. TSH, thyroid stimulating hormone

TSH level	Thyroid density <90HU		Thyroid density >90HU		Total subjects
	n* (4)	%	n* (36)	%	%
TSH <5 (mIU/mL)	1	25.00	3	75.00	4
TSH ≥5 (mIU/mL)	26	72.22	10	27.77	36

**Figure 1:**
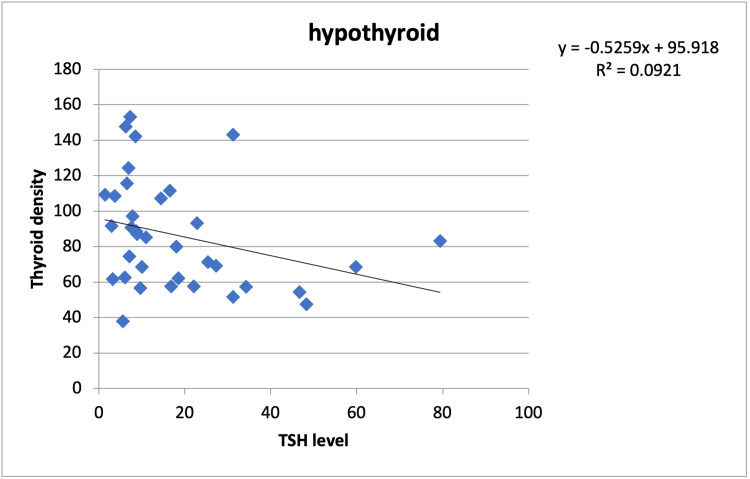
Relationship between thyroid CT densities and TSH levels in hypothyroid TSH, thyroid stimulating hormone; CT, computerized tomography

**Figure 2:**
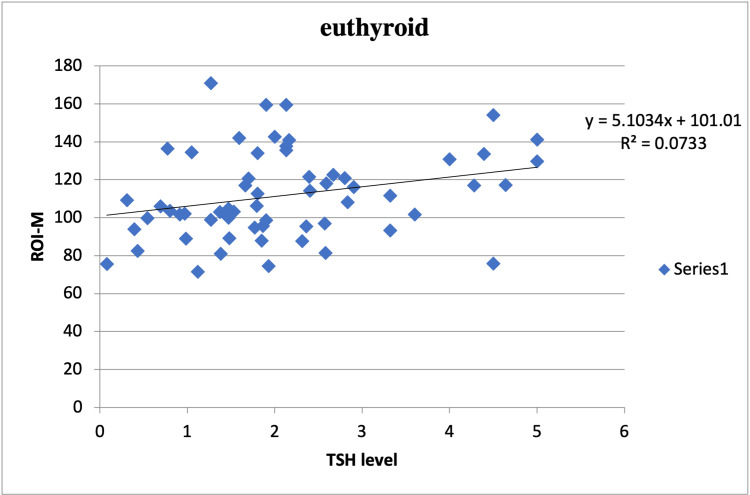
Relationship between thyroid CT densities and TSH levels in euthyroid subjects TSH, thyroid stimulating hormone; CT, computerized tomography

Correlation analysis aimed at elucidating the relationship between TSH levels and thyroid CT densities in both hypothyroid and euthyroid subjects. The results revealed a negative correlation between TSH levels and thyroid CT densities in hypothyroid patients (r=-0.365, p=0.020) and a positive correlation (r=0.270, p=0.036) for euthyroid patients. These findings provide valuable insights into the relationship between TSH levels and thyroid density in the context of thyroid disorders (Figure 1-2).

## Discussion

Our study aimed to establish a meaningful correlation between thyroid density, as measured through NCCT, and serum TSH levels in hypothyroid patients aged 18 years and older presenting with recurrent or chronic respiratory symptoms. Thyroid disorders have a substantial impact on public health, and given the increasing incidence of hypothyroidism, finding reliable and non-invasive diagnostic methods is crucial [1-3].

The majority of subjects in the hypothyroid group (80% or n=48) were identified as housewives. This observation reflects the broader trend of hypothyroidism, which is more prevalent in women, and could be attributed to hormonal and lifestyle factors. In contrast, the euthyroid subjects included in the study were less likely to be housewives (33.33%), highlighting the gender-specific prevalence of hypothyroidism [2,4].

It is noteworthy that the age threshold for inclusion in the study was set at 18 years and above. This decision was made based on the principle that children are more vulnerable to the stochastic effects of ionizing radiation. Consequently, their inclusion might have introduced additional complexities, making the results less generalizable [12,13].

Our analysis revealed a statistically significant difference in the mean thyroid density values between hypothyroid and euthyroid subjects, with a p-value of <0.0001 and a t-value of 5.769, indicating the clear differentiation of these two groups. These results underscore the potential of thyroid density as a valuable diagnostic marker for hypothyroidism when utilizing non-invasive imaging methods like NCCT [11].

The correlation analysis unveiled valuable insights into the relationship between TSH levels and thyroid density. In hypothyroid subjects, we observed a weak negative correlation (r=-0.365, p=0.02), suggesting that as TSH levels rise (indicative of hypothyroidism), thyroid density tends to decrease. Conversely, in euthyroid subjects, we found a weak positive correlation (r=0.27, p=0.036), which implies that as TSH levels remain within the normal range, thyroid density tends to increase. Both of these correlations were statistically significant, further validating the potential diagnostic utility of thyroid density in hypothyroidism.

These findings align with earlier studies, reinforcing the consistency of our results [9-11,14]. For instance, Kaneko et al. reported different CT numbers for thyroid density in patients with various thyroid conditions [14]. Additionally, Kamijo's investigation in 1994 highlighted the significant difference in CT attenuation between normal and diseased thyroid glands [15]. They also found that the serum TSH concentration was highest in patients with the lowest thyroid attenuation, consistent with the manifestation of hypothyroidism [15].

Furthermore, Kaneko et al. demonstrated a direct relationship between CT numbers and the concentration of potassium iodide in phantom experiments [14]. This association suggests that CT numbers are indicative of the iodine concentration within the thyroid tissue, strengthening the premise of using CT density as a diagnostic marker.

In a similar vein, Iida Y et al. conducted a study analyzing thyroid CT numbers and their relationship with iodine concentration [10]. They found substantial variations in CT numbers between normal controls and patients with thyroid diseases. Moreover, they established a significant correlation between thyroid CT numbers and the iodine concentration in the tissue, underscoring the validity of our approach [10,15].

Pandey et al. study, which closely parallels our research, discovered that high TSH groups exhibited considerably lower thyroid CT densities when compared to the normal TSH group [16]. These findings reiterate the potential of thyroid density as an indicator of thyroid functional status [16].

Limitations

The study offers valuable insights into the connection between thyroid density, TSH levels, and the demographic attributes of hypothyroid patients. However, certain limitations must be taken into account. These include a relatively small sample size, skewed gender distribution, age discrepancies, and the potential for occupational bias due to the overrepresentation of housewives. Additionally, the study's reliance on TSH reports from a subset of hypothyroid subjects raises concerns about statistical power. These limitations emphasize the necessity for future research with more diverse, representative samples and a more comprehensive exploration to enhance the understanding of the relationship between thyroid density and TSH levels in hypothyroid patients.

## Conclusions

Our study strengthens the evidence that thyroid density, as measured by NCCT, can serve as a valuable diagnostic tool in identifying hypothyroidism. The correlations between TSH levels and thyroid density further validate this method's potential clinical significance. Our findings contribute to the growing body of research focused on non-invasive and efficient methods for diagnosing thyroid disorders, which can greatly benefit patient care and improve the accuracy and timeliness of diagnoses.
